# Epigenetic Control of Defense Signaling and Priming in Plants

**DOI:** 10.3389/fpls.2016.01201

**Published:** 2016-08-11

**Authors:** Nino A. Espinas, Hidetoshi Saze, Yusuke Saijo

**Affiliations:** ^1^Plant Epigenetics Unit, Okinawa Institute of Science and Technology Graduate UniversityOkinawa, Japan; ^2^Nara Institute of Science and TechnologyIkoma, Japan; ^3^Japan Science and Technology Agency, Precursory Research for Embryonic Science and TechnologyKawaguchi, Japan

**Keywords:** epigenetic control, plant immunity, defense priming, DNA methylation, histone modification, transposable elements, plant-microbe interactions

## Abstract

Immune recognition of pathogen-associated molecular patterns or effectors leads to defense activation at the pathogen challenged sites. This is followed by systemic defense activation at distant non-challenged sites, termed systemic acquired resistance (SAR). These inducible defenses are accompanied by extensive transcriptional reprogramming of defense-related genes. SAR is associated with priming, in which a subset of these genes is kept at a poised state to facilitate subsequent transcriptional regulation. Transgenerational inheritance of defense-related priming in plants indicates the stability of such primed states. Recent studies have revealed the importance and dynamic engagement of epigenetic mechanisms, such as DNA methylation and histone modifications that are closely linked to chromatin reconfiguration, in plant adaptation to different biotic stresses. Herein we review current knowledge regarding the biological significance and underlying mechanisms of epigenetic control for immune responses in plants. We also argue for the importance of host transposable elements as critical regulators of interactions in the evolutionary “arms race” between plants and pathogens.

## Introduction

Plants have evolved sophisticated mechanisms to adapt to fluctuating environments, including immune systems for dealing with diverse infectious microbes that threaten plant growth and survival. In response, plant pathogens have evolved a substantial degree of phenotypic plasticity to avoid and/or suppress recognition by the host (Gomez-Diaz et al., 2012). Such dynamic interactions compel the evolution of plant mechanisms that link pathogen sensing to rapid and effective defense activation to minimize fitness costs.

## Components of the Plant Innate Immune System

Plants have evolved innate immune systems that recognize and respond to pathogens. These systems consist of two tiers of inducible resistance mechanisms, namely pathogen-associated molecular pattern (PAMP)-triggered immunity (PTI) and effector-triggered immunity (ETI) (Chisholm et al., 2006; Jones and Dangl, 2006; Dodds and Rathjen, 2010). PTI represents the first tier of plant immunity and is conferred by pattern recognition receptors (PRRs) that recognize PAMPs or endogenous elicitors, termed damage-associated molecular patterns (DAMPs), generated by pathogen assaults (Boller and Felix, 2009; Macho and Zipfel, 2014; Zipfel, 2014). On the other hand, ETI is typically mediated by nucleotide binding (NB)-leucine rich repeat (LRR) receptors (NLRs) (Takken and Goverse, 2012; Wu et al., 2014; Cui et al., 2015). Plant immunity is characterized by such multilayered structures, which likely enable fine-tuning of defense responses. Fine control of receptor-mediated pathogen recognition and defense signaling downstream of the receptor are fundamental to avoid precocious activation of immune responses that negatively influence plant growth. How do plants mount effective immune response at a minimal fitness cost?

## DNA Methylation: A Dynamic Regulator of Defense Genes

Cytosine methylation of the DNA bases in all sequence contexts, CG and non-CG (CHG and CHH, where H is non-G), is triggered by small interfering RNAs (siRNAs) via a *de novo* methylation pathway termed RNA-directed DNA methylation (RdDM). Canonical RdDM begins by production of RNAs by Polymerase (Pol) IV via NUCLEAR RNA POLYMERASE D (NRPD) subunits, and after several processing steps, the processed RNAs are loaded into ARGONAUTE 4 (AGO4) and base-paired with an RNA scaffold produced by Pol V. Recruitment of AGO4 involves its interaction with NUCLEAR RNA POLYMERASE E1 (NRPE1) of Pol V. Subsequent interaction with DOMAINS REARRANGED METHYLTRANSFERASE (DRM) leads to methylation of DNA target sequences. On the other hand, in the non-canonical Pol II-RDR6-dependent RdDM pathway, Pol II-transcribed single-stranded RNA (ssRNA) is converted into double-stranded RNA (dsRNA) by RNA-DEPENDENT RNA POLYMERASE 6 (RDR6), and then processed into 21–22nt siRNA. The siRNA is loaded into AGO6 that can be directed to the scaffold RNA transcribed by Pol V, which establishes DNA methylation. These methylation marks are maintained through mitosis and meiosis via a pathway catalyzed by METHYLTRANSFERASE1 (MET1) and CHROMOMETHYLASE3 (CMT3) methyltransferases, while REPRESSOR OF SILENCING1 (ROS1), DEMETER-LIKE2 (DML2), and DML3 are DNA glycosylases that dynamically erase DNA methylation via a base excision repair process (details of the RdDM pathway are referred to Law and Jacobsen, 2010; Matzke and Mosher, 2014; Du et al., 2015; Matzke et al., 2015). DNA methylation is a vital process that is also linked to other epigenetic pathways, such as histone methylation and acetylation (Eden et al., 1998; Qian et al., 2012; Du et al., 2015).

Recent studies have extended our understanding of epigenetic control of plant immunity (Alvarez et al., 2010; Sahu et al., 2013; Saijo and Reimer-Michalski, 2013; Ding and Wang, 2015). High-resolution DNA methylation profiling by Dowen et al. (2012) provides the first genome-wide insight into biotic stress-responsive genes in *Arabidopsis*, expression of which is modulated by DNA methylation and demethylation. *met1-3* and *ddc* (*drm1-2 drm2-2 cmt3-11*) plants that are globally defective in maintaining CG and non-CG methylation, respectively, show enhanced defense responses when exposed to *Pseudomonas syringae* pv. *tomato* DC3000 (*Pst*). The same results were obtained in mutants partially defective in CG and non-CG methylation. Moreover, in rice, application of 5-azadeoxycytidine, a DNA demethylating agent, enhances bacterial resistance to *Xanthomonas* (Akimoto et al., 2007). These results are consistent with findings that enhanced RdDM in *ros1–4* plants leads to lowered resistance to *Pst* DC3000 (Yu et al., 2013). In addition, flg22 treatment results in inhibition of transcriptional gene silencing (TGS) as it de-represses RdDM targets. Yu et al. (2013) also confirmed increased bacterial resistance in *ddc* and *met1 nrpd2* plants. *met1 nrpd2* plants also exhibit hypersensitivity response (HR)-like cell death and high *PR1* expression, pointing to de-repression of ETI-like defenses. Furthermore, *ros1 dml2 dml3* (*rdd*) plants, simultaneously disrupted for the three DNA demethylases, show lowered fungal resistance (Le et al., 2014).

Pol V, but not Pol IV, has been implicated in plant immunity (Lopez et al., 2011; Matzke and Mosher, 2014). However, Le et al. (2014) showed an overlap of down-regulated genes between *rdd* and the RdDM mutants, *nrpe1* and *nrpd1*, suggesting that Pol V and Pol IV both regulate defense responsive genes. In addition, fungal infection is enhanced in *nrpe1* and *ago4* plants, while it is slightly reduced in *nrpd1* plants. These results clearly suggest that genome-wide disruption of DNA methylation leads to defense activation, in a way reminiscent of ETI, and that DNA methylation down-regulates immune responses. However, this is not the case for all defense-related genes, as evident in the blast resistance gene, *Pib*, in rice (Li et al., 2011) and in the genome-wide methylation analysis of tobacco plants infected with *Tobacco mosaic virus* (TMV) (Kathiria et al., 2010). Future investigation will be required to determine whether RdDM pathways play a distinctive role in different plant species, between different target genes, against different pathogens or combinations thereof. It is of particular importance to elucidate the regulatory components, the mode of control, and specific target sites in the genome for canonical and non-canonical RdDM pathways in plant immunity, not only in *Arabidopsis* but also in other plant models.

**FIGURE 1:**
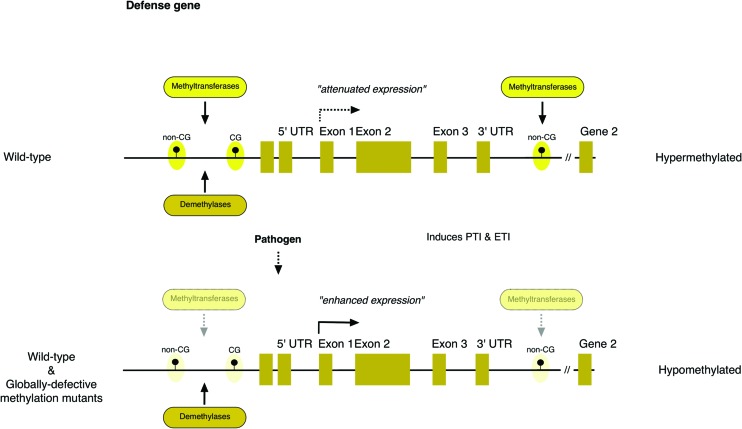
**A general model of epigenetic regulation of defense-related genes.** Hypomethylation of regions flanking both ends of defense-related genes enhances their expression during pathogen challenges. Filled lollipops indicate transposable elements (TEs) or repetitive elements that may be methylated or de-methylated.

These *Arabidopsis* studies also offer insight into methylation states in plant genomes and how changes influence immune responses. In response to *Pst* challenge or flg22 application, DNA methylation levels are globally reduced in all sequence contexts, while the decrease following SA application is restricted to CG and CHG contexts (**Figure 1**). Intergenic transposable elements (TEs) seem to be among the main targets for both canonical and non-canonical RdDM pathways during pathogen challenge. Stress-associated differential methylation in the CG context occurs predominantly ~1 kb upstream of transcriptional start sites (TSS) for protein-coding genes, whereas such methylation in the CHH context occurs high in intergenic regions. Differential methylation in both contexts is over-represented at both ends of protein coding genes. *At3g50480*, a locus encoding a homolog of RPW8 disease-resistance (*R*) protein, undergoes differential methylation changes during pathogen infection. Another *R* gene, *RMG1* (*At4g11170*), is highly induced in response to flg22 and in *met1 nrpd2* plants, while it is compromised in *ros1* plants in which TSS-flanking regions are highly methylated. In *rdd* plants, TEs inserted adjacent to or within 200 bp of promoters and gene bodies, represent major targets of methylation. It is important to note that not only TEs, but also sequences surrounding them are methylated. This is particularly true for those inserted in promoter regions, as shown for *CC-NBS-LRR (At1g58602)* and *jacalin lectin (At5g38550)*. Work on cytosine DNA methylation (mC) in rice and *Arabidopsis* also indicates that proximal regions of TEs, when they are within or in proximity to stress-inducible genes, play a critical role in responsiveness to environmental stress cues (Secco et al., 2015). These findings suggest that regulatory processes modulating methylation at or near gene boundaries, particularly in *R* gene loci, help to fine-tune defense responses, at least in these plant models. Future studies will be required to determine the precise function of these DNA sequences and the molecular mechanisms underlying their recognition and modification.

## Transposable Elements (TEs) in Plant Immunity

A major class of *R* proteins are the NLR immune receptors that mediate ETI to various pathogens. *NLR* genes often form gene clusters in the genome that contain repetitive sequences and TEs (Meyers et al., 2003). The repetitive nature of *NLR*-gene clusters is thought to facilitate rapid expansion and sequence diversification of these genes, possibly by promoting unequal recombination (Friedman and Baker, 2007). It is well documented that TEs inserted in the promoter region often regulate neighboring genes in both animals and plants by changing their epigenetic states (Slotkin and Martienssen, 2007). A recent report shows that TEs in intronic regions can regulate *NLR* expression in *Arabidopsis* (Tsuchiya and Eulgem, 2013). *Arabidopsis RPP7* encodes a CC-NBS-LRR class of NLR that confers resistance to downy mildew, *Hyaloperonospora arabidopsidis* (*Hpa*) (Eulgem et al., 2007). Proper transcription and splicing of *RPP7* requires a protein named ENHANCED DOWNY MILDEW2 (EDM2), which encompasses PHD domains that recognize H3K9 methylation and a putative RNA methyltransferase domain at the *C*-terminus (Lei et al., 2014; Tsuchiya and Eulgem, 2014). In the *edm2* mutant, transcription of *RPP7* is attenuated due to premature termination of the transcripts at the TE within the 1st intron, termed *ECL* (exon 1-containing LTR-terminated transcript). Interestingly, intronic TEs, including COPIA-type retrotransposon in the 1st intron, are targeted by repressive epigenetic marks, such as DNA methylation and H3K9 methylation, as are their intergenic copies, even though they are embedded within the actively transcribed gene unit (Saze et al., 2013; Tsuchiya and Eulgem, 2013). Maintenance of repressive epigenetic marks in intronic TEs seems to be important for proper expression of *RPP7*, since RPP7-mediated ETI to *Hpa* is impaired in plants deficient for H3K9 methylation, recapitulating the immuno-compromised phenotype of *edm2* plants. Similarly, reduced DNA methylation in *DECREASE IN DNA METHYLATION1* (*DDM1*) mutants or *CMT3* results in a transcription defect of *RPP7* (Le et al., 2015). Interestingly, even though *RPP7* shows sequence polymorphism among different *Arabidopsis* accessions due to TE insertions within intronic regions (Tsuchiya and Eulgem, 2013), most of these natural accessions harbor the COPIA element in the 1st exon. This implies that TE insertion has selective advantages, possibly by providing a fine-tuning mechanism for *RPP7* expression (McDowell and Meyers, 2013). As reported, epigenetic states of TEs are dynamically altered in response to biotic stress (Dowen et al., 2012). Epigenetic control of intragenic TEs may thus act as a regulatory mechanism for *NLR* gene expression in plant-pathogen interactions.

## Histone Modification and its Role in Systemic Acquired Resistance

Defense activation at recognition sites for PAMPs or effectors generates and delivers systemic signals throughout the plant, which result in enhanced immunity to a broad spectrum of pathogens, called systemic acquired resistance (SAR) (Conrath, 2011; Fu and Dong, 2013; Kachroo and Robin, 2013; Conrath et al., 2015). During and after SAR, defense-related genes become sensitized to subsequent pathogen attack at distal, non-challenged sites, known as defense priming. Defense-primed plants are enabled to mount a swift defense response, which involves “kick starting” of up-and down-regulation for priming target genes.

Among potential mechanisms underlying defense priming, histone modifications are of particular interest since they affect the landscape of transcription of defense-related genes through evolutionarily highly conserved functions (Waterborg, 2011). Recent studies in plants have implicated H3K4me3, H3K4me2, H3K9ac, H4K5ac, H4K8ac, and H4K12ac in defense priming. In particular, H3K4me3 is considered a primary chromatin marker of stress memory (Conrath et al., 2015). Recent studies on heat stress acclimation in *Arabidopsis* present a model in which transient binding of the heat-inducible transcription factor HsfA2 leads to sustained H3K4 methylation and thus the maintenance of heat stress memory, i.e., acquired thermotolerance (Lamke et al., 2016). Notably, HsfA2 function is dispensable for the acquisition of thermotolerance *per se*, but indispensable for its maintenance (Charng et al., 2007). On the other hand, Mozgová et al. (2015) have shown that the histone chaperone, CAF-1, mediates a repressive chromatin state of defense genes, by retaining nucleosome occupancy and suppressing H3K4me3 marking. However, loss of CAF-1 alone is insufficient to activate SA-related defense genes. These findings suggest that CAF-1-conditioned chromatin modification prevents inappropriate defense activation. Further investigation will be required into the mechanisms by which defense signaling triggered upon pathogen recognition overcomes this barrier and leads to a priming state, partly through increasing H3K4me3 deposition, at both challenged and non-challenged sites.

Histone acetyltransferases (HATs) and deacetylases (HDACs) also participate in control of defense priming. *hac1-1* (histone acetyltransferase 1) plants are compromised in bacterial resistance and defense priming following PTI (Singh et al., 2014). This is the first evidence that an HAC1-dependent pathway is responsible for defense priming after exposure to recurring abiotic stress cues. HAC1 does not seem to direct resistance to *Pst per se*, suggesting that HAC1 links recurring stress response activation to defense priming. It remains to be shown how HAC1 establishes the epigenetically primed states at open chromatin target sites. Consistent with a positive role for histone acetylation in defense activation, loss of HDA19 results in de-repression of SA-based defenses (Choi et al., 2012) and depletion of the HDAC HDT701 enhances H4 acetylation and resistance to both fungal and bacterial infection (Ding et al., 2012).

It has been reported that defense priming and these histone marks are transgenerationally inherited (Heard and Martienssen, 2014; Iwasaki and Paszkowski, 2014; Kinoshita and Seki, 2014; Crisp et al., 2016). A recent study in yeast has proven for the first time that H3K9 methylation is heritable over several generations (Audergon et al., 2015). Given the evolutionary conservation for functions of these histone marks, it is conceivable that histone modifications provide a basis for heritable immune response memory.

**FIGURE 2:**
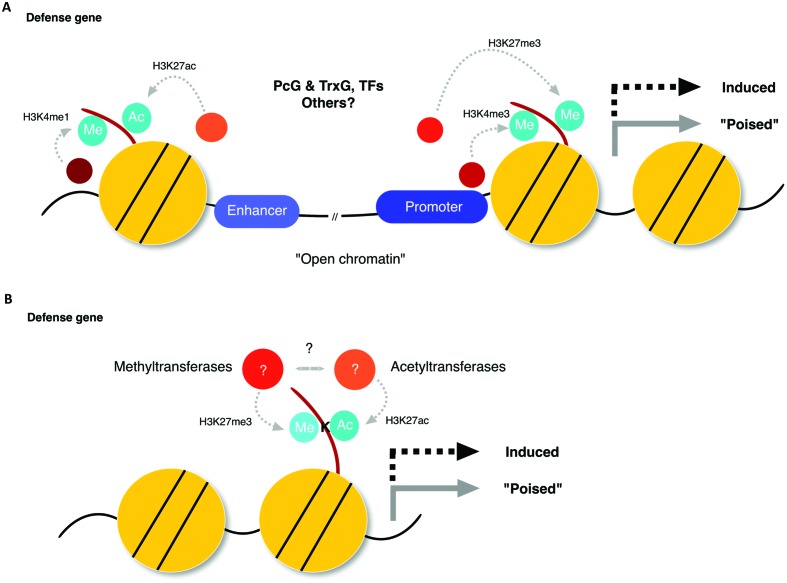
**Bivalent phenomena hypothesis in plant defense priming. (A)** An interplay of opposing histone modification marks in enhancer and promoter regions modulate the expression status of defense-related genes. Polycomb-group (PcG) and Trithorax (TrxG) proteins may assemble with interacting proteins, such as transcription factors (TFs). **(B)** Opposing histone marks on the same lysine-site (*K*-site) act as a switch to modulate the expression status of defense-related genes.

A subset of, if not all, defense genes activated in SAR, seems to be primed as a consequence of interplay between different histone modifications, via mechanisms that are still poorly understood (Conrath, 2011; Gutzat and Mittelsten Scheid, 2012; Spoel and Dong, 2012; Saijo and Reimer-Michalski, 2013; Conrath et al., 2015; Ding and Wang, 2015). Priming of defense-related genes has a fitness advantage compared to their substantial activation (van Hulten et al., 2006). It is tempting to speculate that this has contributed to the evolution of genomic regions that undergo histone modifications to establish such a priming state at target genes, which enables effective transcriptional reprogramming toward enhanced resistance in response to second challenge. In animals, enhancer and promoter sites are often marked with H3K4me1/H3K27ac and H3K4me3/H3K27me3, respectively (Azuara et al., 2006; Bernstein et al., 2006; Zhou et al., 2011; Calo and Wysocka, 2013; Voigt et al., 2013) (**Figure 2**). These combinatorial histone marks can occur in a gene-autonomous manner, and seem to exert complex regulatory effects, as is the case of H3K4me3/H3K27me3 in the promoter region (called a bivalent promoter) (Bernstein et al., 2006). It should be noted, however, that bivalency is not restricted to narrow genomic regions, as enhancers can influence target genes as much as a million bases distant (Pennacchio et al., 2013). Thus, cautions need to be taken when considering bivalency, which can occur at the same nucleosome unit harboring two antagonizing marks in different histone molecules or in one histone molecule (e.g., H3K4me3/H3K27me3 in promoters; **Figure 2A**), or in separate nucleosome units (e.g., H3K27me3/H3K27ac in promoters and enhancers, respectively; **Figure 2B**). In acclimation to abiotic stress, an increase of transcription-permissive H3K4me3 occurs when plants are exposed to recurring stress cues without removing transcription-repressive H3K27me3 (Saleh et al., 2007; Avramova, 2015). Given that not only pathogen recognition, but also adverse abiotic conditions can induce defense priming in plants (Singh et al., 2014; Vivancos et al., 2015), it is of high interest to test whether bivalent histone modification also plays a role in defense priming. Future studies will be required to clarify the functional significance of bivalent modification, which may be distinct from that of either transcription-permissive or -repressive modification alone.

## Summary and Future Perspectives

In this review, we have integrated recent advances in epigenetic control of defense-related transcriptional reprogramming and priming.

Earlier findings about the role of DNA methylation in modulating defense responses (Pavet et al., 2006; Luna et al., 2012) and recent epigenome analysis (Dowen et al., 2012; Yu et al., 2013; Le et al., 2014) have revealed the importance of fine control of DNA methylation near the boundaries of defense-related genes, repetitive sequences, and TEs during immune responses in plants. It is still unclear whether methylation patterns established in the host genome reflect specific infection strategies and/or infection states of pathogens. If this is the case, however, it predicts that pathogen-induced changes in DNA methylation status, possibly at specific genomic sites, can be sensed by a surveillance system in the host. In this context, RdDM components may act as part of such immune sensory systems, much as alterations in RdDM efficiency following pathogen challenge lead to hypomethylation of defense-related genes and to immune activation.

Histone acetylation and methylation have emerged as critical regulators of defense priming. These modifications occur at specific histone residues in concert with or as a consequence of transcriptional reprogramming in response to pathogen challenge or environmental cues, which result in sustainable reconfiguration of the nucleosome. It is also interesting to see whether and how histone acetylation and methylation are established and coordinated with each other during defense activation and priming. At present, we know little about histone modifications of target regions in the genome, the dynamic changes they induce, and histone-modifying enzymes involved in plant immunity. Findings for HAC1-mediated priming, for instance, provide a good start toward a deeper understanding of the significance and modes of actions of these histone modifications in plant-pathogen interactions. Moreover, these efforts need to be integrated with elucidation of the signals that link pathogen recognition to epigenetic modifiers in both cell-autonomous and non-cell autonomous contexts (as reviewed in Spoel and Dong, 2012; Conrath et al., 2015). Emerging data suggest common adaptive strategies in plant acclimation to different biotic/abiotic stressors, which seem to involve cooption of evolutionarily conserved epigenetic regulation in a manner unique to plants.

Last but not least, it should be noted that our review particularly focuses on DNA methylation, TE control and histone modification in the contexts of defense-related gene expression, NLR receptor expression and SAR/priming, respectively, do not indicate restriction of these epigenetic controls to the corresponding aspects of plant immunity. These and other epigenetic mechanisms may play a role in fine control of different steps in plant immunity, and thereby contribute to its multilayered structure.

## Author Contributions

NE conceptualized the contents of the manuscript, wrote, and edited the manuscript. HS wrote the manuscript. YS wrote, edited the manuscript, and directed the contents of the manuscript.

## Conflict of Interest Statement

The authors declare that the research was conducted in the absence of any commercial or financial relationships that could be construed as a potential conflict of interest.
